# Mechanically Stretchable and Electrically Insulating Thermal Elastomer Composite by Liquid Alloy Droplet Embedment

**DOI:** 10.1038/srep18257

**Published:** 2015-12-16

**Authors:** Seung Hee Jeong, Si Chen, Jinxing Huo, Erik Kristofer Gamstedt, Johan Liu, Shi-Li Zhang, Zhi-Bin Zhang, Klas Hjort, Zhigang Wu

**Affiliations:** 1 Department of Engineering Sciences, Uppsala University, Box 534, SE 751 21, Uppsala, Sweden; 2 Department of Microtechnology and Nanoscience (MC2), Chalmers University of Technology, Kemivägen 9, SE 412 96, Gothenburg, Sweden; 3 State Key Laboratory of Digital Manufacturing Equipment and Technology, Huazhong University of Science and Technology Luoyu Road 1037, Wuhan, 430074, China

## Abstract

Stretchable electronics and soft robotics have shown unsurpassed features, inheriting remarkable functions from stretchable and soft materials. Electrically conductive and mechanically stretchable materials based on composites have been widely studied for stretchable electronics as electrical conductors using various combinations of materials. However, thermally tunable and stretchable materials, which have high potential in soft and stretchable thermal devices as interface or packaging materials, have not been sufficiently studied. Here, a mechanically stretchable and electrically insulating thermal elastomer composite is demonstrated, which can be easily processed for device fabrication. A liquid alloy is embedded as liquid droplet fillers in an elastomer matrix to achieve softness and stretchability. This new elastomer composite is expected useful to enhance thermal response or efficiency of soft and stretchable thermal devices or systems. The thermal elastomer composites demonstrate advantages such as thermal interface and packaging layers with thermal shrink films in transient and steady-state cases and a stretchable temperature sensor.

The novel functionalities of soft materials as being stretchable and compliant, may trigger a new generation of smart devices and systems that can be conformally attached onto complex surfaces or have dynamic configurations^1^,^2^. With soft materials, new device concepts for wearable electronics and soft robotics have recently shown numerous possibilities in many applications such as in sensors^3^, actuators^4^, power sources^5^, optical devices^6^, wireless components^7^ and biomedical devices^8^. To support these smart systems, new soft materials, which can adapt to different applications or situations and be concurrently processed in simple and robust ways, are in great demand. Among them, polymer-based composites with functional additives represent the most promising one. So far, highly stretchable polymer composites^9^,^10^,^11^, electrically conductive composites^12^,^13^,^14^, highly dielectric composites^15^ and several other functional composites^4^,^16^,^17^,^18^ have been reported for stretchable electronics and soft actuators.

As one of the potential areas for thermally controllable devices or systems can provide new functions for stretchable technology. From this perspective, soft or stretchable, thermally responsive and programmable materials are of high potential in, e.g., temperature sensors^19^, heaters^20^, coolers, thermoelectric transducers, thermal actuators^4^ and high frequency wireless components. However, thermally conductive soft or stretchable materials, which are simultaneously electrically insulating suitable for device fabrication, are not widely studied yet.

Polymer based thermally conductive composites have been studied for thermal interface materials (TIMs) or packaging materials due to their conformality on complex surfaces. The compliant coverage on any surface offers high thermal contact conductance^21^,^22^,^23^,^24^,^25^ and hence high heat dissipation with increased efficiency for the devices or systems. Apart from this thermal enhancement, polymer-based composite materials can provide lightweight and simple applicable processing. For instance, polymer-based TIMs accompanied with their processing techniques have been investigated in highly thermally conductive polymers^26^,^27^ and composites^28^,^29^,^30^,^31^,^32^,^33^,^34^,^35^ in order to minimise thermal contact resistance at the interface as well as to have a high thermal conductivity. But they still suffer from difficulties in material preparation processes or implementation methods when applied to a real device to achieve expected properties^26^,^36^. More importantly, stretchability of these materials is rarely reported or insufficient for real applications. In addition, electrically insulating TIMs or packaging materials in electronic components are preferred in order to avoid current leakage or electrical shorts. Insulation is important for safety issues in cases such as high current wires, high frequency antennas and high speed microprocessors at interconnect sides of electronic devices.

Here, we report a thermal elastomer composite (TEC), which is mechanically stretchable, thermally conductive, electrically insulating, and easily processable without a high demand on equipment or laboratory infrastructure, Fig. 1(a). Moreover, its conformality minimises thermal contact resistance leading to maximization of heat flux through interfaces in both transient and steady-state configurations. To provide compliance and deformability, gallium-based liquid alloy (Galinstan) droplet^37^,^38^,^39^ fillers are introduced in the polydimethylsiloxane (PDMS) elastomer matrix in this work. The liquid-state metallic droplet fillers offer conformal and stretchable features together with relatively high thermal conductivity, while the PDMS matrix contributes with mechanical formation, electrical insulation and mechanical stretchablility. The mechanical, thermal and electrical properties of the TECs are investigated with regard to various aspects at different weight fractions of the liquid alloy fillers. Simple thermoplastic actuations on the TECs at different temperature conditions are demonstrated with improved thermal conduction at transient states as well as steady states. This TEC is compatible with and adaptable to current technology, such as soft actuators and stretchable sensors^40^,^41^,^42^. It can provide new possibilities for wearable electronics and soft robotics as well as for conventional rigid body systems with complex surfaces, such as TIMs or soft thermal packaging materials.

## Results

### Overview

**Figure 1:**
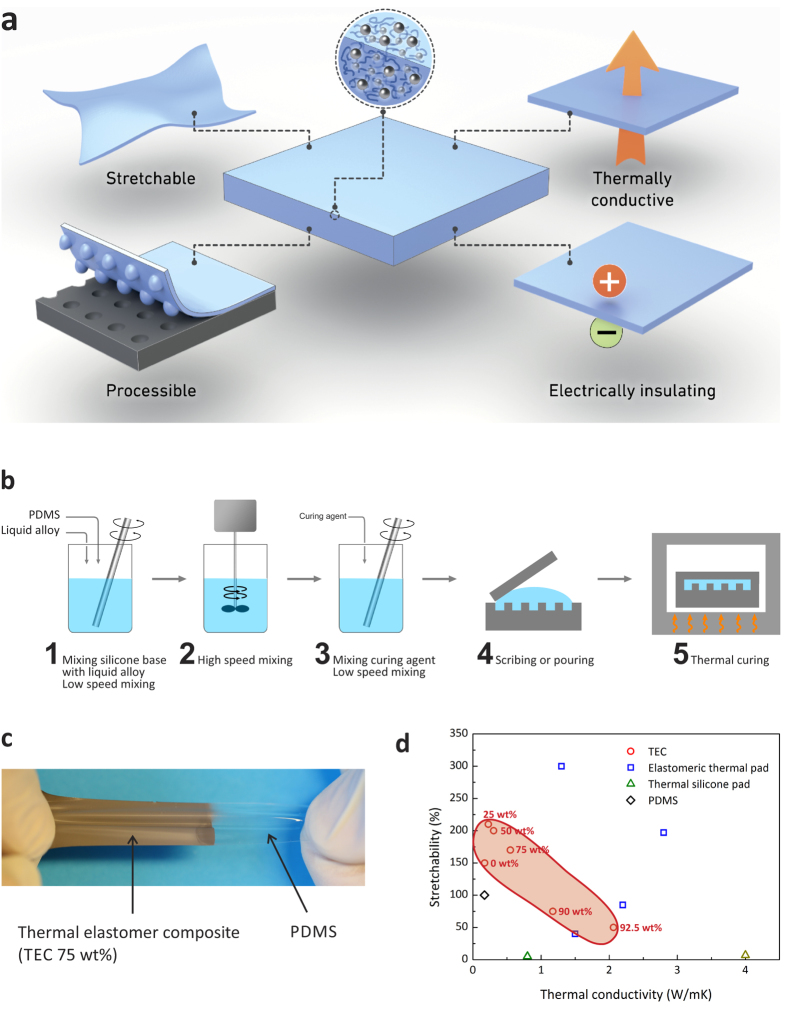
Stretchable thermal elastomer composite (TEC). (**a**) Schematic illustration of the concept of a liquid alloy droplet embedded elastomer composite for high heat conduction and insulating with a soft, stretchable behaviour, (**b**) preparation process steps of the thermal elastomer composite, (**c**) a cured TEC (75 wt%) film of a 50 μm thickness which was stretched by hand, and (**d**) thermal conductivity vs. maximum stretchability of TECs compared to the reported works and commercialised product (SARCON, Fujipoly) of electrically insulating polymer based thermal conductive materials.

The liquid alloy was mixed with a silicone–based elastomer by a simple mechanical mixing process, Fig. 1(b). High speed mixing is advantageous in dispersing the liquid alloy as small droplets embedded inside the silicone base (Low speed mixing would not yield droplet sizes desired for this purpose.). These small droplets kept their morphologies after being immersed into the silicone base as well as in the cured elastomer matrix. Homogeneously dispersed liquid alloy fillers in smaller sizes could give more stable mechanical behaviour at high strains and higher thermal conductivity by narrowing the distance among droplets in the PDMS network. A processed TEC film under tension by hand stretching is shown in Fig. 1(c). Commercially available, electrically insulating and thermally conductive polymers are compared with our TECs of different compositions regarding stretchability and thermal conductivity in Fig. 1(d). The TECs show a large window of tunable properties and only a few competitive materials that are stretchable, thermally conductive and electrically insulating could be found.

### Processability

The processability was tested in several cases, e.g., spinning, laminating and casting (for more details, see Figure S1 in the Supplementary Information, SI). Micrometer scale structures were successfully shaped with common laboratory tools, such as a spin coater, a film applicator and a microchannel mold. The processability of the mixtures of the liquid alloy and the PDMS base was comparable to that of pure PDMS that is widely accepted as an easily processable material. The mixtures showed a conformal behaviour to a structured surface before curing and resulted in a lowered thermal contact resistance in a device with a simple implementing process. At high weight fractions of liquid alloy fillers, *e.g*. 90 and 92.5 wt%, the mixtures became highly viscous and did not flow on surfaces of a petri-dish, a silicon wafer, a glass slide, a PET film or a copper foil. Consequently, the mixtures with such high fractions were laminated or scribed by applying pressure using a film applicator or a slide glass on a target surface, instead of using spin coating or gravimetric flowing to prepare thin layer structures. Having moderate viscosity, and being thermally curable and processable in similar ways as PDMS, this simplifies the implementation of a TEC for a large area, conformal and thermally conductive layer or for a complex surface structure. In the case of the mixtures of 25 wt% and 50 wt% liquid alloy fillers, the curing rates of the mixtures became faster than that of PDMS. However, over 50 wt%, the curing time became longer than for PDMS. The TEC with 75 wt% liquid alloy fillers could be successfully casted into micro channels from a microfluidic channel mold that had a minimum of 25 μm wide channels, Figure S1(c) in the SI.

Good adhesion between the TEC (75 wt%, hereafter, the specified percentage in blanket informs weight percentage of liquid alloy fillers in a PDMS matrix.) and PDMS is necessary to fabricate device structures, and this was tested with a bonded film structure of two materials after curing. The sample was not broken at the interface until it was broken (Figure S2 in the SI). Before and after stretch cycling, the adhered interfaces of the TEC and PDMS in a double layer structure were observed with field emission scanning electron microscopy (FE-SEM) (Figure S3 in the SI).

### Microstructures

**Figure 2:**
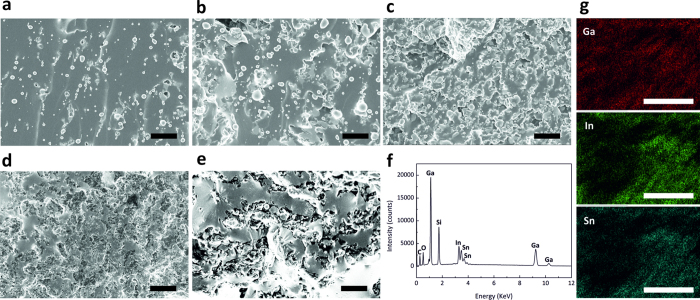
Microstructure of the TECs, investigated with SEM, EDX and AFM. FE-SEM images of different weight fractions of liquid alloy fillers with (**a**) 25 wt%, (**b**) 50 wt%, (c) 75 wt%, (**d**) 90 wt%, and (**e**) 92.5 wt%. The scale bars indicate 10 μm (**a**–**e**), (**f**) EDX spectrum and (**g**) element map of the cross section of the composites of 75 wt% liquid alloy fillers (the scale bar indicates 10 μm).

The liquid alloy droplets were observed in the PDMS matrix with FE-SEM, Fig. 2 (a–e). The droplet sizes of the liquid alloy ranged from 2–3 μm to 50 nm on the fractured surface (Figure S4 in the SI). As expected, the amount of droplet fillers was increased with increasing the fraction of the liquid alloy in the composite. Small voids inside the composites after curing can affect the properties, i.e. by reducing the liquid alloy compactness, degrading the mechanical performance and decreasing the thermal conductance by hindering thermal transport. To minimise the density of voids, air bubbles in the mixture were removed before molding and curing by storing the mixture at –20 °C for 6 hours in a fridge to delay the mixture curing for waiting for the air bubbles to be floated up to the surface. In the TECs with the liquid alloy fraction below 75 wt%, air bubbles floated to the surface and could be omitted since the viscosity of the mixtures was low enough. However, at higher weight fractions of liquid alloy fillers, pressurised laminating or casting had to be used in order to minimise voids. For a TEC, the shape of the liquid alloy droplet fillers and the contact interface between liquid alloy fillers and the PDMS matrix can significantly influence heat transfer over the filler gaps and thus the thermal conductivity. To achieve better adhesion at the interface between the liquid alloy fillers and the PDMS matrix, an additional buffer interface material^43^ or surface functionalization^44^ can be introduced. This should be subjected to a further investigation in the future. Energy dispersive X-ray spectroscopy (EDX) data show distribution of droplet fillers that were homogeneously dispersed in the PDMS matrix, Fig. 2(f,g).

### Mechanical characterization

**Figure 3:**
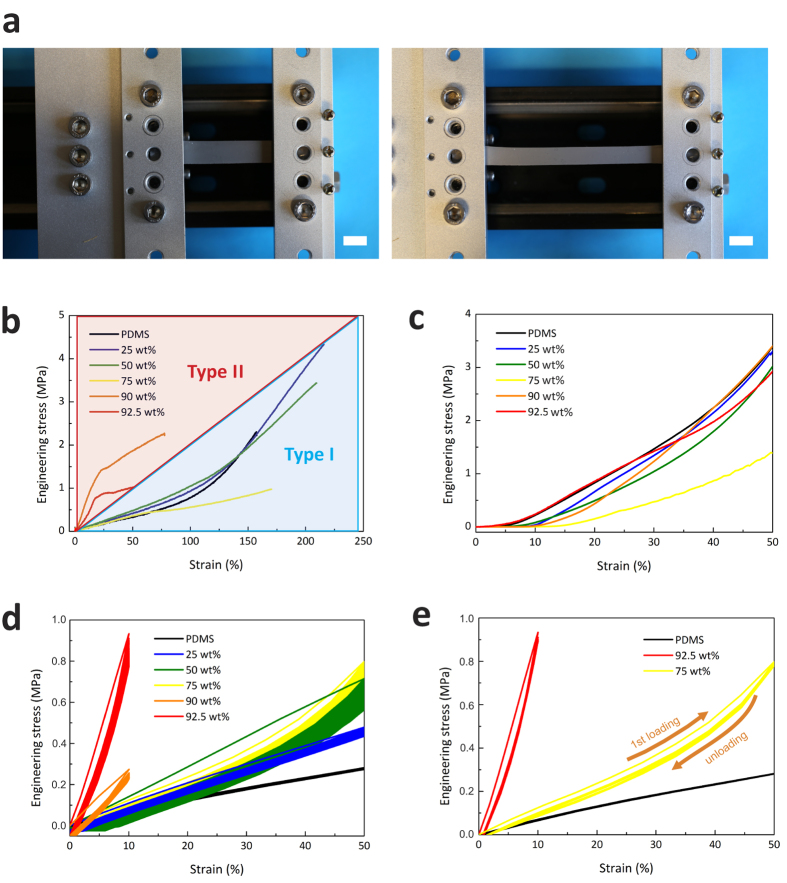
Mechanical properties of the TEC with different weight fractions of the liquid alloy fillers. (**a**) a stretched thermal elastomer composite film of 75 wt% liquid alloy fillers in a mechanical strain setup without strain (left) and with 100% strain (right), (**b**) stress-strain curve from tensile tests, (**c**) from compressive tests, (**d**) cycling tests of loading and unloading 2,000 times and (**e**) the initial 5 cycles of the TEC (90 wt%) under the 50% strain condition.

The photograph in Fig. 3(a) depicts a stretched TEC sample of 75 wt% liquid alloy fillers with the strain (engineering strain) of 100%. According to their mechanical and thermal properties as well as their processabilities when varying the filler’s weight fractions, the TECs can be categorised as Type I or Type II, as shown in Fig. 3(b). From the tensile tests, we observed that the liquid alloy embedment in PDMS does not affect the mechanical properties of the TEC in Type I much compared to the native PDMS^45^. The liquid alloy droplet fillers follow the PDMS matrix deformation due to their liquid characteristics, which helps the stretchability, conformality and softness of the composite. The TECs in Type I follow the expected stress-strain curve and hysteresis of conventional elastomers. Young’s moduli of the TECs in Type I show similar levels as that of PDMS, Fig. 3(b,c).

Above a threshold around 75 wt% liquid alloy fillers, the liquid fillers changed the stress-strain response significantly. In the higher weight fraction cases (Type II), the stress-strain curve shows a ductile behaviour in the high strain region with a softening (yielding) point. Irreversible deformations occurred when the strain was higher than the yielding point in Type II, which can originate from damages in the polymer network under high strains. As shown in SEM micrographs, the liquid alloy fillers tended to be connected over the threshold in Type II. This percolation threshold makes a big difference in the mechanical behaviour of TECs. Moreover, the rule of mixture based on composite mechanics could not be applied to the mechanical behaviour of a liquid alloy embedded elastomer composite because TECs had liquid fillers instead of solid fillers. Further theoretical investigations and further experimental studies are necessary to reveal the mechanism of this phenomenon.

Briefly, there is a transition region with the TEC properties between the fraction of 75 wt% and 90 wt% liquid alloy fillers. Type I is more stretchable than Type II, and having lower viscosity it has better processability to fabricate micrometer scale structures. On the other hand, Type II shows lower mechanical stretchability and higher viscosity, indicating a relatively lower processability than that of the PDMS. Depending on the target application, the mechanical behaviour of TECs can be tuned only with a liquid alloy filler fraction. All measured properties of the tested TECs are listed in Table 1.

Cyclic tensile tests of TECs entailed 2,000 cycles at 50% strain for Type I and 10% strain for Type II. In the case of the Type II, large mechanical hysteresis was observed during the first mechanical cycling, Fig. 3(d). After cycling, the length of the specimen was recovered to its original length with no remaining irreversible plastic deformation. The stress-strain hysteresis of the TECs became larger along with filler fraction increases and the hysteresis was reduced during the cycling, Fig. 3(e). There was no observed change in the stress-strain relations of TECs with different strain rates (Figure S5 in the SI).

### Thermal characterization

**Figure 4:**
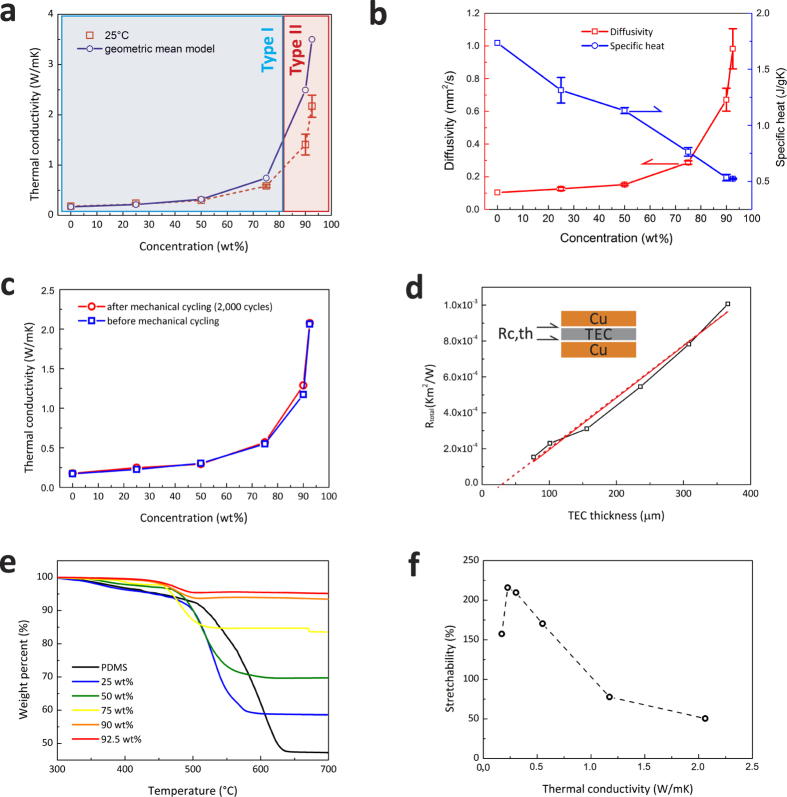
Thermal properties of the TECs at different weight fractions. (**a**) thermal conductivity compared to a theoretical estimation, (**b**) thermal diffusivity and specific heat capacity, (**c**) thermal conductivities before and after mechanical cycling of 2,000 times with 50% strain (Type I) and with 10% strain (Type II), (**d**) thermal contact resistance of the TEC (75 wt%) layer measured by the xenon flash method, (**e**) thermal stability of TECs of different weight fractions of liquid alloy fillers and (**f**) a relation of mechanical stretchability and thermal conductivity of TECs under different fractions.

By introducing the liquid alloy that has a high thermal conductivity, 16.5 W/m·K^46^, compared to that of PDMS (0.15–0.2 W/m·K)^47^, the thermal conductivity of the composite was considerably increased while maintaining the high stretchability. Furthermore, the thermal properties of the TECs are tunable by controlling the weight fraction of the liquid alloy fillers, although there is a trade-off between mechanical and thermal properties as well as processability. By increasing the weight fraction of liquid alloy fillers, the thermal conductivity of the TEC was dramatically increased at around the 75 wt% liquid allot filler fraction. The TEC (92.5 wt%) has 12 times higher thermal conductivity compared to PDMS, Fig. 4(a). The threshold showing the significant jump in thermal conductivity is positioned between the fractions of 75 wt% and 90 wt% liquid alloy fillers, corresponding to the mechanical properties. This phase inversion in the composite seems to correspond to a percolation threshold of mechanical and thermal properties. The thermal conductivity of the TECs was maintained with a small variation when changing the temperature^48^. The process reliability of the high speed mixing process for TECs was proven with the variation of the thermal conductivity from three different batches as shown in Fig. 4(a,b). This was calculated from the density (Figure S6 in the SI), the diffusivity and the heat capacity at 25 °C.

Assuming that the fillers are homogeneously distributed and their shape is spherical, the effective thermal conductivity of TEC can be estimated by the rule of mixture using a geometric mean model as follows^49^,


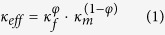


where *κ*_*eff*_*, κ*_*f*_, and *κ*_*m*_ are the thermal conductivity of a composite, a filler and a polymer matrix, respectively, and *φ* is the volume fraction of the fillers. The calculated values are plotted together with the measured data in Fig. 4(a), which is supported by a previous work^50^. The thermal diffusivity of the TECs increased with increasing the weight fraction of liquid alloy fillers, which helps a transient thermal response, Fig. 4(b). The specific heat capacity of the TECs decreased with increasing the weight fraction of liquid alloy fillers due to the lower heat capacity of the liquid alloy (256 J/kg·K)^46^ compared to that of PDMS (1,460 J/kg·K)^47^. The thermal conductivity of the TECs could be systematically improved by increasing the percentage of liquid alloy in the PDMS, which caused the density and thermal diffusivity to increase, and the heat capacity to decrease concurrently. Referring to Fig. 4(c), the thermal conductivity of the TECs remained unchanged before and after the mechanical cycling of 2,000 times at a strain rate of 100 mm/min. In-plane thermal conductivity of the TEC (75 wt%) was 0.6 W/m·K, which indicates homogeneity in the thermal conductivity of the TEC.

The thermal contact resistance of the TECs to a copper surface was measured by the xenon flash method, Fig. 4(d), to test the performance of the TECs as a TIM layer. The thermal contact resistance of the TEC (75 wt%) was compared with a soldered sample with an SAC (Sn-Ag-Cu) alloy as a reference (Figure S7 in the SI). The extrapolated intercept value on the Y-axis (thermal resistance) yields a very small thermal contact resistance with the TEC, which is estimated by:


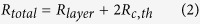


where *R*_*layer*_ is the thermal resistance of the TEC layer and *R*_*c,th*_ is the thermal contact resistance at the interface between the TEC and the substrate. The TEC (75 wt%) had a very low thermal contact resistance close to zero, which is comparable to the reference sample (SAC alloy) in the same sample structure. Again, the TEC shows excellent wetting and conformality on the surface and it is possible to have high-efficiency heat transfer by decreasing thermal contact resistance.

Thermo-gravimetric analysis (TGA) indicated the thermal stability of TECs up to 300 °C, which is comparable to PDMS, Fig. 4(e). The thermal decomposition rates were well matched with the densities of TECs with the different weight fractions of the liquid alloy fillers. The weight fraction of the liquid alloy fillers (that is a liquid state from −19 °C to 1,300 °C) corresponded to the weight change of TECs after decomposition of the PDMS matrix at 300–400 °C.

Figure 4(f) shows the relationship between the maximum stretchability and thermal conductivity of the TECs investigated in this work. By adding more liquid droplet fillers, the thermal conductivity of the TECs is increased while the mechanical strength and stretchability are degraded. Nonetheless, Type I TEC could be stretched over 100% of the initial length, Fig. 3(a), which was higher than the stretchability of the PDMS.

### Electrical characterization

The TECs were electrically insulating before and after curing due to the elastomer matrix encapsulating the liquid alloy fillers. The uncured silicone base has a relatively low surface energy while the liquid alloy has a much higher surface tension. Hence, the liquid alloy tends to stay completely separated and isolated as micro- and nano-sized droplets if there is not much liquid alloy volume fraction in the elastomer matrix. As shown in Fig. 2, liquid alloy fillers began to be connected to each other in the case of Type II. The electrical resistivity of the TECs was examined with a two-point probe measurement technique. The TECs showed insulating properties before and after curing as well as before and after mechanical cycling. These properties were estimated with a maximum readable resistance in the multimetre (maximum resistance, 50 MΩ) that gave a resistivity of more than 0.5 GΩ·cm. In the case of the TEC (92.5 wt%), it has a higher probability to link liquid alloy fillers over the elastomer matrix, which may make it electrically conductive when compressed. But this must be highly related to the uniformity of droplet sizes and spatial distribution. A high voltage breakdown test is needed to understand dielectric strength for high power device applications.

### Application demonstrations

**Figure 5:**
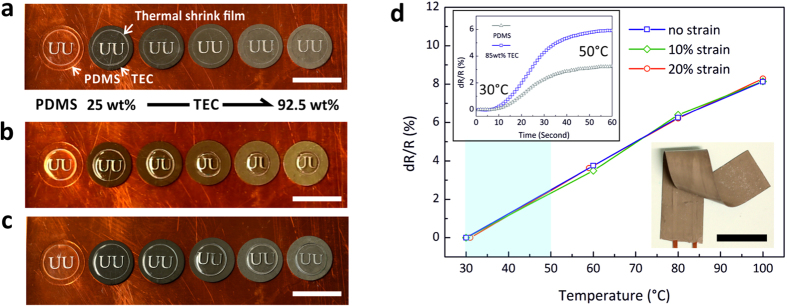
Thermal shrinkage of thermoplastic films (**a-c**) and a stretchable temperature sensor packaged with the TEC (**d**). (**a**) Original status of thermal shrink films on the TECs and PDMS, (**b**) transient shrunk status by different time responses of thermal shrink films differentiated by different TECs after 7.5 minutes at 150 °C, (**c**) fully shrunk status of thermoplastic films on different TECs after 20 minutes at 140 °C and (**d**) the resistance change curve of the stretchable RTD packaged in the TEC and the transient thermal response of the sensors in the different packages (inset). The scale bars indicate 20 mm.

Thermal shrink films were used to demonstrate how the thermal diffusivity and thermal conductivity of the TEC layers of different weight fractions change the thermal response and efficiency, Fig. 5. The thermal shrink films on different TEC layers shrank with different time responses on a 1 mm thick copper plate placed on a hot plate, Fig. 5(a). Under sufficiently high heat flux through the TEC layers to the thermal shrink films at 150 °C, the TECs of higher weight fractions led to faster shrinkage due to their higher thermal diffusivity as well as its higher thermal conductivity, Fig. 5(b). When heated further at 150 °C, all of the thermal shrink film samples shrank to the same size. On the other hand, at 140 °C, which did not provide enough heat flux to the thermal shrink films through the TEC layers, the shrink film shrunk much more on the TEC layers of higher thermal conductivity due to their higher thermal conductivity, Fig. 5(c). In all cases, no more shrinking was observed after further heating at 140 °C.

Stretchable liquid alloy RTDs (Resistance Temperature Detector) were made in the original PDMS or a TEC (85 wt%) package, Fig. 5 (d). The TEC (85 wt%) had an acceptable processability for fabrication of the liquid alloy RTD structure, which had high thermal conductivity and stretchability together. It had a TCR (Temperature Coefficient of Resistance) of 0.0011 Ω/Ω/ °C, which was tested under relaxed and 20% stretched conditions. The TEC packaged liquid alloy resistor showed its stretchable function and reliable RTD response at different temperatures and applied strains. The TEC packaging showed no degradation after many stretching cycles and the liquid alloy RTD worked without large variations when being relaxed or under strain. Owing to the higher thermal conductivity of the TEC packaging, the sensitivity of the RTD was improved. The time constant was 30 s for both types of packaging. Due to variations in the spray process, the resistance values of the stretchable RTD packaged in the TEC and the PDMS were 13.3 Ω and 7.3 Ω at 25 °C without strain, respectively.

## Discussion

According to our observations, high speed mechanical mixing is essential for breaking down the liquid alloys into small sized droplets in order to make electrically insulating composites. Mixing can be further optimised by mixing time, speed, head design, or by using different preparation methods, such as layer-by-layer process or controllable process of liquid alloy filler shapes. The microstructures of the TECs can be controlled for a better uniformity of droplet sizes and spatial distribution to reach higher thermal conductivity and more uniform characteristics of the TECs. The leakage of liquid alloy droplet fillers from a PDMS matrix in high weight fractions under a high pressure should be taken into account for a contact surface material with the TEC to prevent an alloying reaction of liquid alloy droplets. A barrier layer such as graphene or tungsten can be used to prevent this^51^,^52^.

The thermo-mechanical behaviour at high temperature conditions and under dynamic pressure or stretching is worth a further study for a dedicated application. In addition, the gallium oxide layer persistently present on the liquid alloy droplets, known as oxide skin, can be further investigated to improve the mechanical and thermal characteristics of the TECs. An inert and oxide-free surrounding condition will help to have less oxide skin layers forming on the droplets. This may lead to higher thermal conductivity and lower mechanical stiffness of the composites.

In conclusion, the liquid alloy embedded elastomers, two-phase composites, have been shown to possess several advantages. TECs are: (1) soft and stretchable comparative with PDMS; (2) highly thermally conductive than PDMS and tunable in thermal conductivity; (3) conformal on complex surfaces, which can enhance thermal contact conductance with the high thermal conductivity; (4) electrically insulating before and after stretching, similar to PDMS; and finally, (5) easily processable like PDMS with simple and cost-effective processing steps for microscale structuring and large area coatings.

With simple processing steps, our mechanically stretchable, thermally conductive and electrically insulating composites, TECs, display high potential for use as not only heat conductors, heat spreaders at interfaces and packaging layers, but also active materials in thermally programmable and tunable actuators that have curvilinear or complex morphology. For example, stretchable thermoelectric generators or coolers will require TECs as a packaging material for increasing energy conversion efficiency. In addition, the thermal management with the TEC in stretchable devices including heat sources will be improved and get a wider operation window, higher efficiency as well as longer life time and better stability of the device. To further improve the functionality of TECs, understanding of phonon transfer^53^ through the interfaces of the liquid alloy droplets and the polymeric chains of PDMS is required. Stretchable thermal energy conversion devices and soft thermal actuators will work more efficiently and with higher performance when the TECs are used.

## Methods

### Composite Preparation

Firstly, the liquid alloy (Galinstan, Geratherm Medical AG) and the silicone base of the PDMS (Elastosil RT 601, Wacker Chemie) were mixed with the liquid alloy by manual stirring. Secondly, a machining tool (Model 398, Dremel) assembled with a brush head (EZ473SA, Dremel) was used for high speed mixing at switch setting 10 (7,000 – 10,000 rpm) for 10 minutes. The mixture was then cooled down in ambient room temperature for 30 minutes. Thirdly, the curing agent was added to the mixture at 18:1 ratio by hand mixing. The mixture was poured into a petri-dish and was placed in a freezer at –20 °C for 6 hours to remove the air bubbles inside the mixture. Finally, the mixture was cured in an oven at 75 °C for 14 hours. (For more details, refer to Materials and Experiments section in the SI)

### Characterizations

The microstructure of the TECs with different weight fractions of liquid alloy fillers was observed with field emission scanning electron microscopy (FE-SEM, Leo 1550, Zeiss). For mechanical property measurements, the cured composites on a petri-dish mold or on a glass substrate were cut into dog bone shaped specimens with a stencil mask and a knife. Stress-strain curves of the TECs were obtained by a tensile test machine (AGS-X, Shimadzu). Cyclic tests of TEC samples were performed in a different tensile test machine (Microtester 5548, Instron) with the same strain rate. For thermal diffusitivity measurements, TEC samples (Figure S8 in the SI) with different weight fractions of the liquid alloy fillers were prepared. The strain was calculated as elongation length divided by the initial gauge length (i.e. engineering strain) and the stress was calculated as the measured force divided by the cross section area of the neck of the specimen. The xenon flash method (LFA 447, Netzsch) was used for thermal diffusivity measurements of the TECs. The reference sample, Pyroceram 9606, was used for heat capacity calculations in the xenon flash software (Nanoflash). The measurement variation of the xenon flash method is shown in Figure S9 in the SI. The density was measured with Archimedes’ principle by using a density measurement kit (YDK01, Sartorius) using a balance (SBC22, Scaltec). The thermal stability of the TECs was examined using the TGA method (Pyris 1, PerkinElmer). The thermal contact resistance was measured with a sandwich structure of three layers, where a TEC layer was positioned between two copper plates with different thicknesses, while a commercial solder paste (SAC305, SnAgCu alloy) served as reference. The xenon flash machine (LFA 447, Netzsch) was used for thermal contact resistance measurement. The electrical conductivity was tested with a multimetre (Fluke 77, Fluke).

### Thermal shrink films on TEC layers

Thermal shrink films (Krympplast transparent, Panduro Hobby) were cut with a cutting plotter (CraftRoboPro, Graphtec) and TEC layers (260 μm thickness) were punched with a round shape cutter (17.5 mm diameter). Thermal shrink films were placed on TEC layers on a hot plate and they were observed with a DSLR camera (EOS 6D, Canon). Temperature conditions were set at 140 °C to test effects of thermal conductivity of the TECs and at 150 °C to test effects of thermal diffusivity of the TECs.

### Stretchable RTDs

The stretchable RTD was fabricated with a liquid alloy conductor pattern (350 μm width and 190 mm length, 8.5 × 16.0 mm in the pattern of the sensing area) in the TEC (85 wt%) package (thickness 500 μm). The liquid alloy circuit was patterned with a spray process using a 9 μm thick copper stencil mask^7^,^54^, which was prepared by transferring a laser printer (Aficio MP C4501, RICOH) printed pattern on a water soluble adhesive paper (Toner transfer paper, Pulsar Professional) to the copper foil with a hot laminator (Star photo laminator PL714, Peach) followed by iron chloride etching at 75 °C. For one side etching, one side of the copper foil was covered with a paper tape (Conform, Rtape corp.) during the etching process.

The stretchable RTD was characterised with a K-type thermocouple connected to a thermometer (Fluke 561 IR thermometer, Fluke) on a hot plate (RCT Basic, IKA Werke) and a multimetre (34405A, Agilent Technologies) connected to a computer. Seven pieces of slide-glass were positioned on the temperature sensor during the temperature sensor evaluation to make a good thermal contact between the sensor and the hot plate. The strain in the device was maintained by fixing it with adhesive tapes on the hot plate. For the time constant measurement, a one millimeter thick TEC and PDMS layer were put under the RTDs to distinguish time-dependent signal changes of RTD by different packages

## Additional Information

**How to cite this article**: Jeong, S. H. *et al.* Mechanically Stretchable and Electrically Insulating Thermal Elastomer Composite by Liquid Alloy Droplet Embedment. *Sci. Rep.* **5**, 18257; doi: 10.1038/srep18257 (2015).

## Supplementary Material

Supporting Information

## Figures and Tables

**Table 1:** Properties of the TECs of different weight fractions of the liquid alloy fillers.

Sample weight fraction [wt%]	Sample weight fraction [vol%]	Density^a^ [g/cm^3^]	Thermal diffusivity^b^ [mm^2^/s]	Specific heat capacity^b^ [J/g·K]	Thermal conductivity^b^ [W/m·K]	Young’s modulus [MPa]	Ultimate elongation [%]	Electrical resistivity [GΩ·cm]
PDMS	0	1.02	0.10	1.7	0.17	0.65	150	>0.5
25	5.0	1.32	0.11	1.5	0.22	0.81	210	>0.5
50	13.7	1.72	0.15	1.1	0.30	0.95	200	>0.5
75	32.2	2.64	0.29	0.76	0.58	0.72	170	>0.5
90	58.7	3.79	0.67	0.53	1.4	7.4	75	>0.5
92.5	66.1	4.18	0.98	0.52	2.2	3.3	50	>0.5

^a^Averaged values of the measured samples from 5 batches.

^b^Averaged values of measured samples from 3 batches; All data is measured and calculated at 25 °C.
