# Supplementary material for: Mechanically Stretchable and Electrically Insulating Thermal Elastomer Composite by Liquid Alloy Droplet Embedment
Source: Sci Rep. 2015 Dec 16;5:18257. doi: 10.1038/srep18257 (PMC4680911; doi:10.1038/srep18257)

Supplementary Information

Mechanical Stretchable and Electrically Insulating Thermal Elastomer Composite by Liquid Alloy Droplet Embedment

Seung Hee Jeong, Si Chen, Jinxing Huo, Erik Kristofer Gamstedt, Johan Liu, Shi-Li Zhang, Zhi-Bin Zhang, Klas Hjort and Zhigang Wu*

Correspondence and requests for materials should be addressed to Z.G.W. (email: [Zhigang.Wu@angstrom.uu.se](mailto:seunghee.jeong@angstrom.uu.se)).

**Materials and Experiments**

For a better understanding of the TECs, various weight fractions (25, 50, 75, 90 and 92.5 wt%) of the liquid alloy in PDMS were processed to measure mechanical, thermal and electrical properties and to examine processability.

*Preparation process of thermal elastomer composite*: First, with weighing the corresponding amount of the liquid alloy (Galinstan, Geratherm Medical, AG) and the silicone base of the PDMS (Elastosil RT 601, Wacker Chemie) to the designed weight fractions, the silicone base of the PDMS was mixed with the liquid alloy by stirring with a glass rod in a cup for 2 minutes by hand in order to break the liquid alloy into droplets and immerse them into the silicone base. In the next step, a machining tool (Model 398, Dremel) assembled with a brush head (EZ473SA, Dremel) was used for high speed mixing at switch setting 10 (7,000 – 10,000 rpm) for 10 minutes. To prevent unexpected quick curing by adding the curing agent at a too high temperature which is generated during the high speed mixing, the mixture was cooled down to room temperature for 30 minutes before the curing agent of the PDMS was added. Afterwards, the curing agent was added to the mixture at a ratio of 18:1 (18 of the weight of the silicone base weight and 1 of the weight of the curing agent, all the fractions presented in the composite are given by the ratio of liquid alloy and the PDMS base, disregarding the curing agent fraction) by hand mixing with a glass rod for 2 minutes. The mixture was poured into a Petri dish and was placed in a freezer at -20 °C for 6 hours to remove the air bubbles inside the mixture. Finally, the mixture was cured in an oven at 75 °C for 14 hours. The sample that had the 95 wt% was not cured even though a longer curing time of one week and the higher fraction of the curing agent (9:2 mixing ratio) were applied in an oven at 75 °C.

*Observation of microstructures of the composites*: The microstructure of the TECs with different weight fractions of the liquid alloy fillers was observed with field emission scanning electron microscopy (FE-SEM, Leo 1550, Zeiss). The cross sections of the samples were prepared from the tensile tested specimens in order to avoid touching the liquid droplet fillers during cutting. The cross section may have a different appearance but this method was the best condition that we found from trying several other methods such as cutting and tearing to make cross sections of the composites. The cross sections of the composites were coated with palladium by sputtering before SEM inspections.

*Measurement of tensile and compressive behaviour of the composites*: The TEC samples for mechanical property measurements, which were cured on a Petri dish mold or on a glass substrate, were cut into dog bone shaped specimens with a hard mask and a knife. The cutting was done without stopping during the cutting to avoid edge cracks or discontinuities along the specimen edge line. A straight gauge volume, that is length, thickness and width, was measured with a micrometer before testing. Stress-strain curves of the TECs were obtained by a tensile test machine (AGS-X, Shimadzu) and the stroke rate was 100 mm/min. The compression test was done at 1 mm/min rate. Cyclic tests of the TEC samples were done in a different tensile test machine (Microtester 5548, Instron) with the same strain rate. The TECs of Type I were stretched to 50% and Type II were stretched to 10% during the cycling, considering the maximum stretchibility. The sample was mounted in the test setup clamps (the cross-heads) with PDMS blocks as a spacer between the sample and the heads to avoid stress concentration near the edges of the heads. A small slip from the mounting heads during the test was also minimized with the PDMS blocks. The engineering stress was calculated from the load divided by the original cross-sectional area and the engineering strain was calculated as the elongation divided by the original gauge length. Young’s Modulus was fitted to the measured stress-strain curve within 50% strain in case of Type I and within 10% strain in case of Type II.

*Measurement of thermal diffusivity*: The TEC samples (**Figure S8** in the SI) for the thermal diffusivity measurements were prepared with different weight fractions of the liquid alloy fillers. The mixture of the liquid alloy and PDMS was poured into a mold (Petri dish) and cured in an oven by controlling its thickness at 1 mm. For the case of higher weight fractions (Type II), the samples were laminated with a glass scriber on a glass substrate with a spacer structure to control the height of the samples. To minimize voids inside the mixture before curing, the prepared samples were stored in a freezer for 6 hours at -20 °C and they were cured in an oven at 75 °C for 14 hours. (For lower weight fractions below 50 wt%, vacuuming made a quick curing of the mixture due to a catalytic mechanism of the liquid alloy fillers, such as platinum in PDMS and heating originated from the friction of air bubbles in PDMS. For high weight fractions, the curing speed became slower than for the low weight fraction cases.) After curing, they were cut into a circular shape of 12.7 mm diameter for the xenon flash method (LFA 447, Netzsch) and then the samples were coated with graphite on both sides, using a graphite spray (Graphit 33, Kontakt Chemie). The reference sample, Pyroceram 9606, was used for heat capacity calculations in the xenon flash software (Nanoflash). The thermal conductivity, *κ* of the TEC was calculated by *κ=ρC_p_D* where *ρ* is the density, *C_p_* is the specific heat capacity and *D* is the thermal diffusivity of the TEC obtained from the xenon flash method. The measurement variation of the xenon flash method is shown in **Figure S9**.

*Measurement of density*: The density of the TECs was measured with the same samples as used for the thermal conductivity measurements, before graphite coating, with a density measurement kit (YDK01, Sartorius) using a balance (SBC22, Scaltec). The weight of the TECs was measured in air and in water for calculation of the density, using Archimedes’ principle.

*Measurement of thermal stability*: The thermal stability of the TECs was examined using the TGA method (Pyris 1, PelkinElmer). The tests were performed from 300 °C to 800 °C with a temperature ramping of 5 °C/min.

*Measurement of thermal contact resistance*: A sandwich structure of three layers, which was a TEC layer between two copper plates (12.7 mm diameter and 1 mm thickness), was prepared with 12.7 mm diameter TECs of different thicknesses. Both sides of the copper plates were polished and cleaned with acetone, IPA and DI water with sonication before structuring the samples. Reference samples, which were soldered with a commercial solder paste (SAC305, SnAgCu alloy), were prepared with different thicknesses and the same structure and by the same processing as the TEC samples. Both sides of all samples were coated with graphite. The xenon flash machine (LFA 447, Netzsch) was used for thermal contact resistance measurement.

*Measurement of electrical resistivity of the composites*: The electrical conductivity of the TECs was tested with a multimeter (Fluke 77, Fluke). The sample area was 10 × 10 mm and the sample thickness was 1 mm. Liquid alloy electrodes were coated on both sides of the sample.

**Reference**

[1] Wu, C.–L., Lin, H.–C., Hsu, J.–S., Yip, M.–C. & Fang, W. Static and dynamic mechanical properties of polydimethylsiloxane/carbon nanotube nanocomposites. *Thin Solid Films*, **517**, 4895 (2009).

[2] Johnston, I. D., McCluskey, D. K., Tan, C. K. L. & Tracey, M. C. Mechanical characterization of bulk Sylgard 184 for microfluidics and microengineering*. J. Micromech. Microeng.* **24**, 035017, (2014).

[3] ASTM D5470-12, Standard Test Method for Thermal Transmission Properties of Thermally Conductive Electrical Insulation Materials, ASTM International, West Conshohocken, PA, 2012 Source: DOI: 10.1520/D5470-12

Figure S1. Processability of the thermal elastomer composite shown with (a) a prepared mixture before curing, (b) cured samples with different weight fractions of the liquid alloy droplet fillers, (c) a spin coated composite layer with 100 µm thickness on a silicon wafer, (d) a laminated composite film of 50 µm thickness with a film applicator on a slide glass, (e) a micro-channel structure casted from an SU-8 mold and (f) a stretched and rolled TEC of 50 µm thickness.


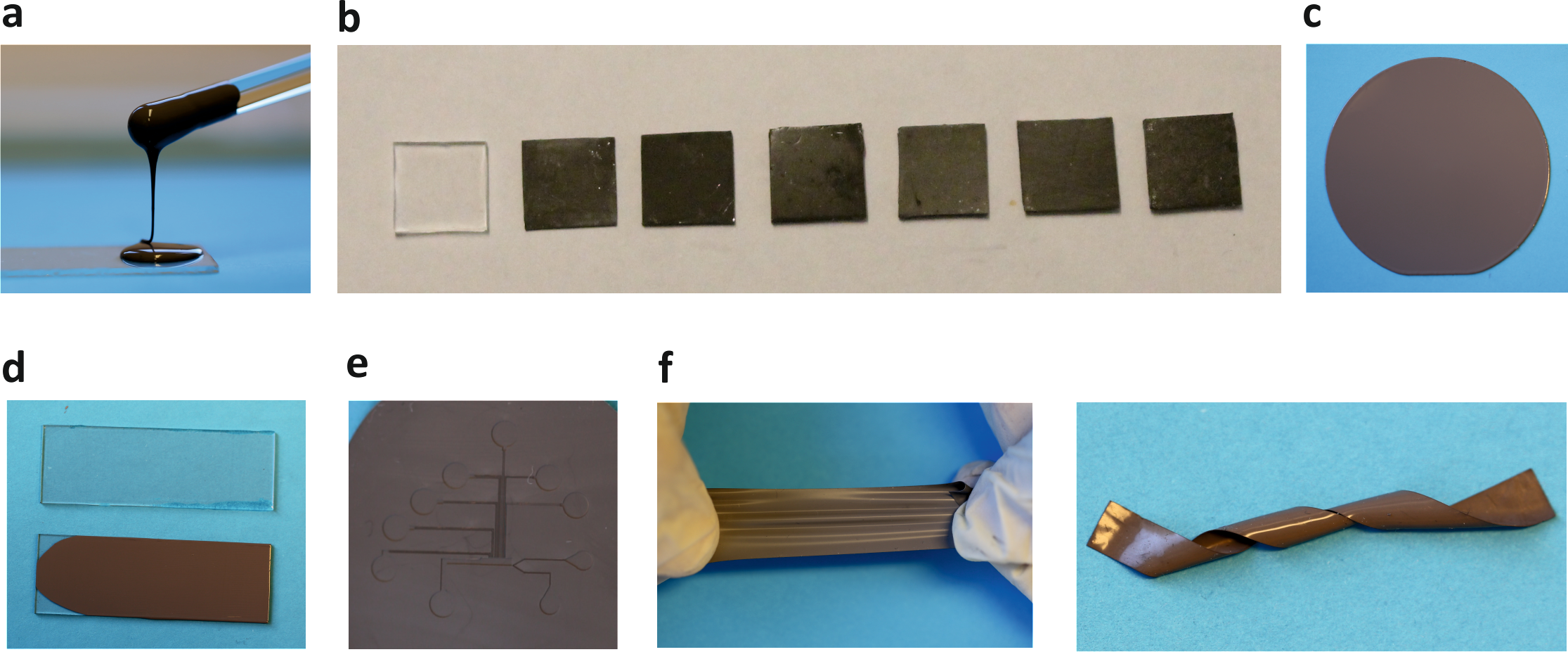


Figure S2. An adhesion test sample (a) of the TEC (75 wt%) bonded to a PDMS film, laterally and (b) a stretched sample.


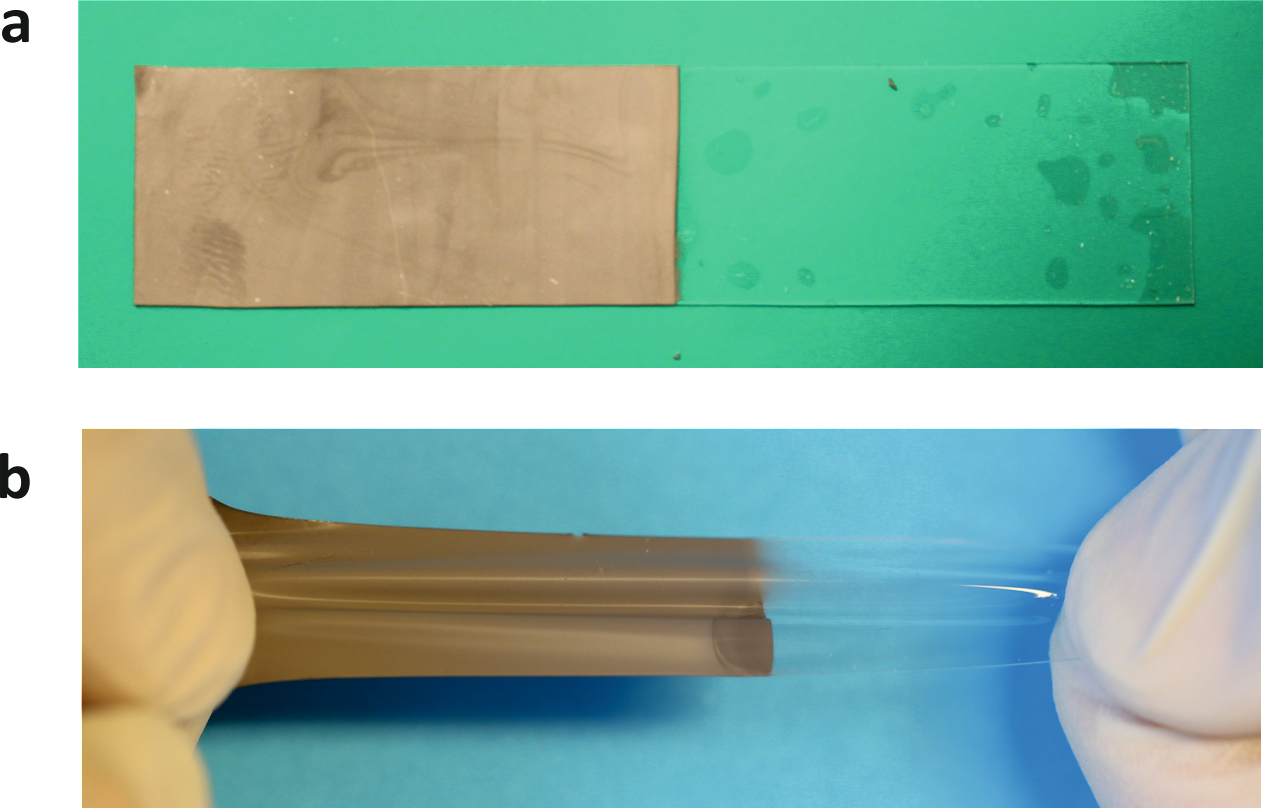


Figure S3. An SEM images of the bonded interface of the TEC (75 wt%) to the PDMS layer. The interface between two materials (a,b) and the magnified cross sections of the TEC (c,d) before stretching. The SEM images of the interface of PDMS-TEC before (e) and after 1,000 times stretch cycling (f). The scale bar indicates 100 µm in (a), 10 µm in (b,c) and 1 µm in (d).


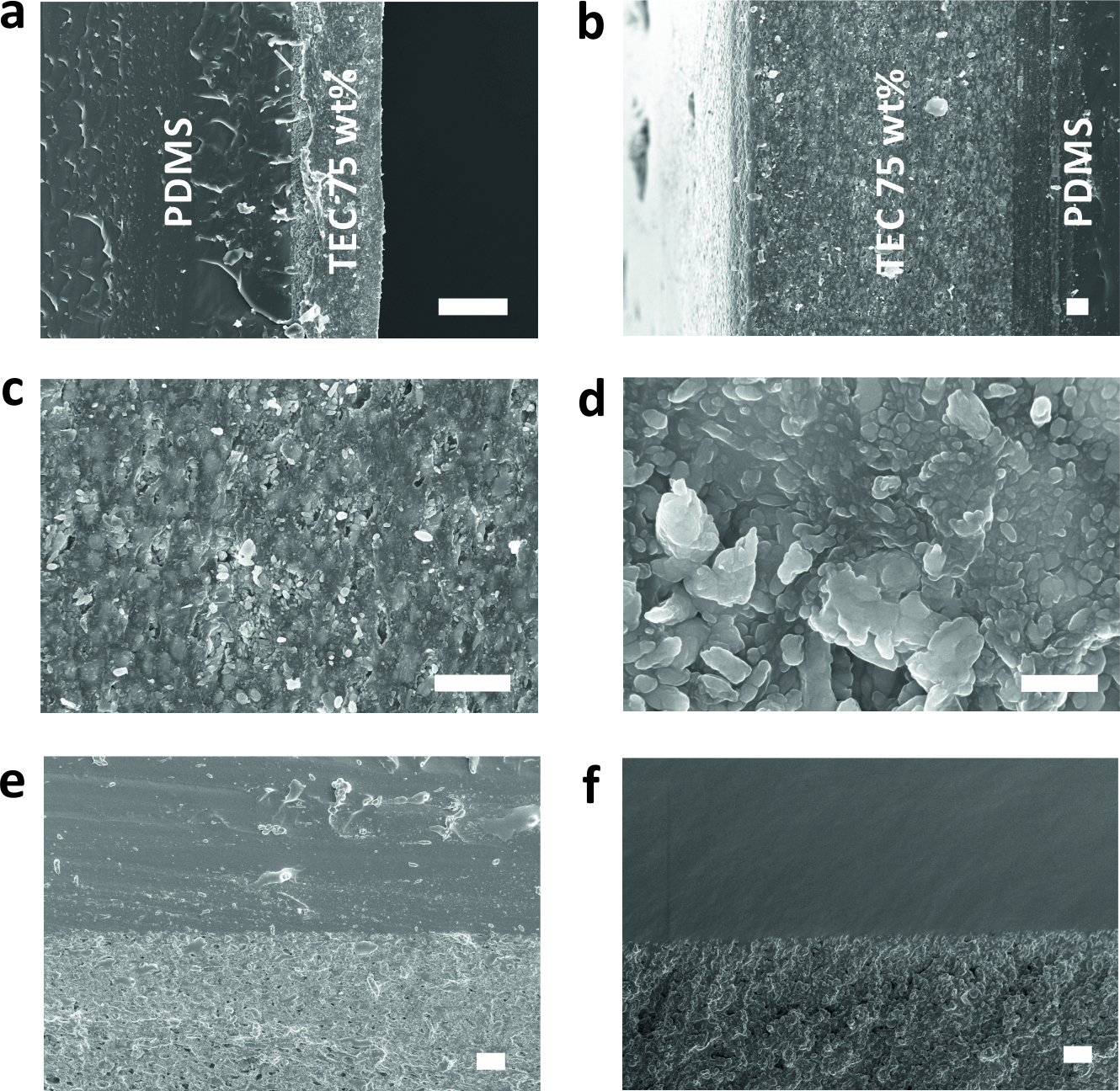


Figure S4. An SEM image of the TEC with high magnification investigations. The scale bar indicates 1 µm.


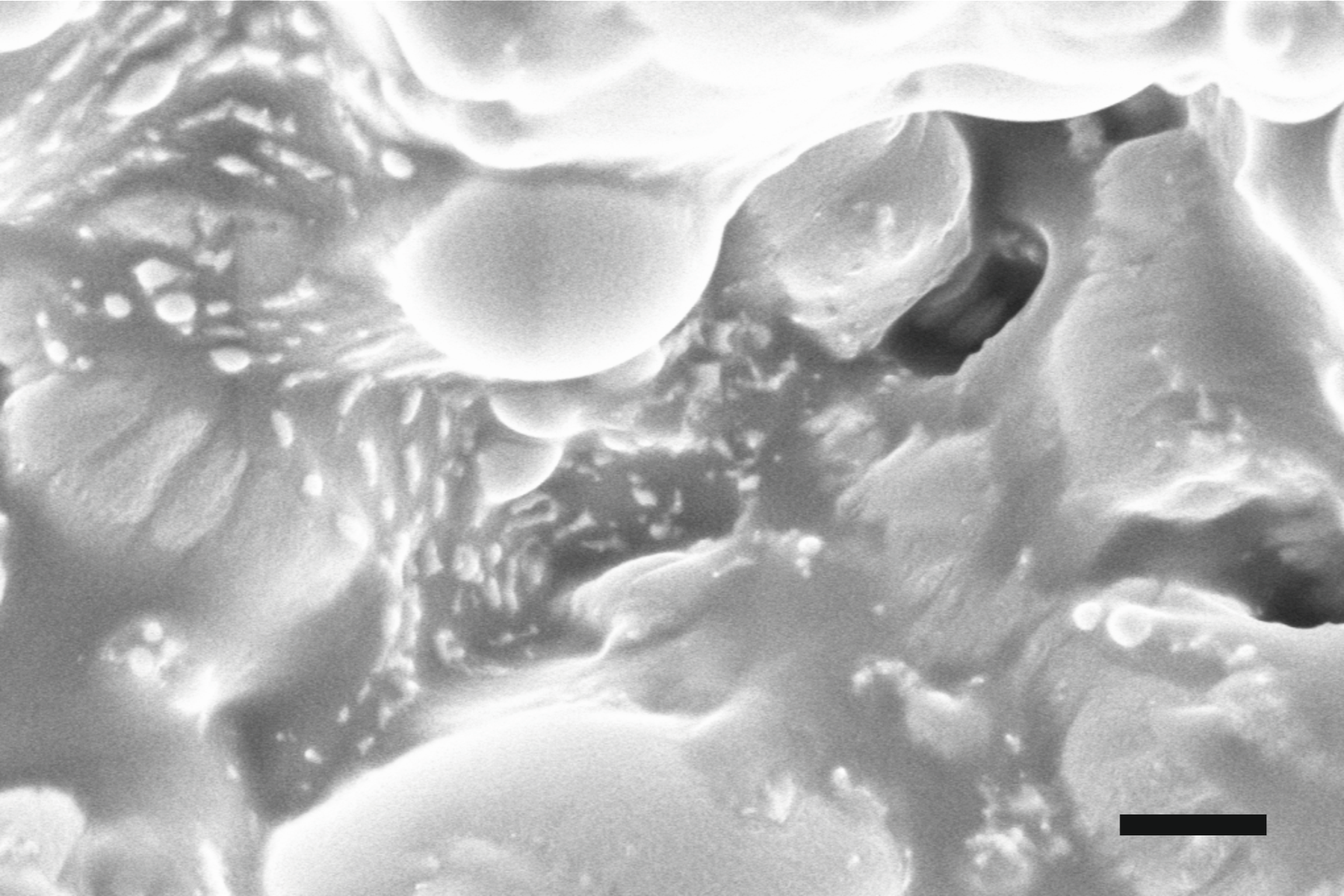


Figure S5. The stress-strain curves of 10 cycles of a TEC (75 wt%) under the different strain rates (a,b) and the mechanical cycling tests of TECs (c).


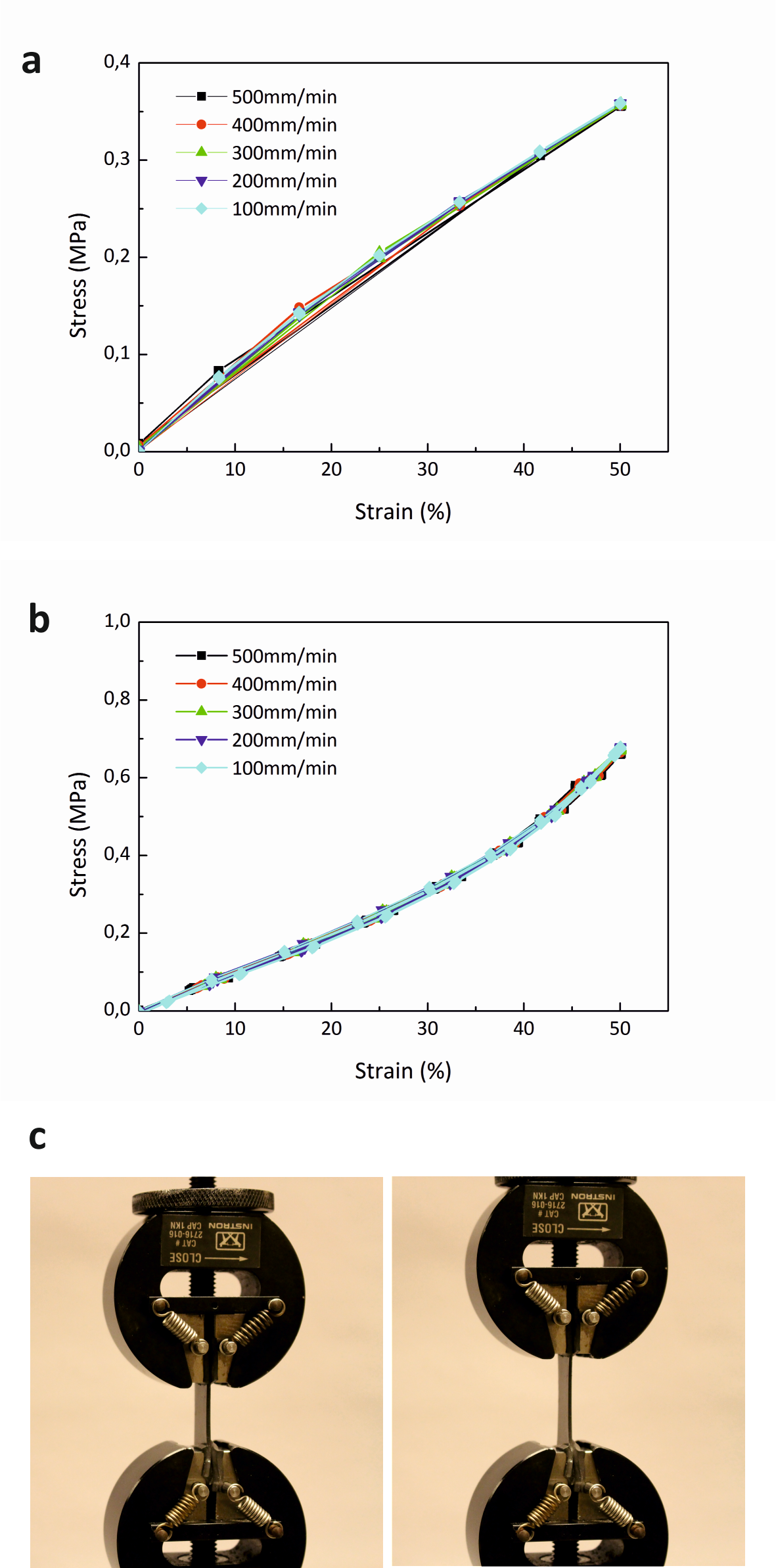


Figure S6. The density of the TEC samples.


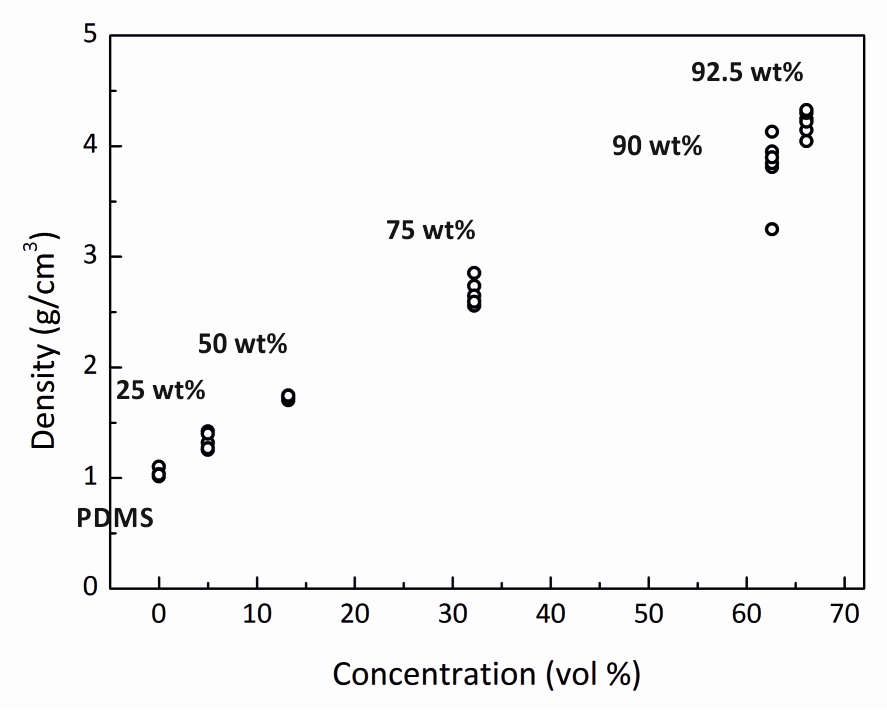


Figure S7. Thermal contact resistance measured by the Xenon flash method.


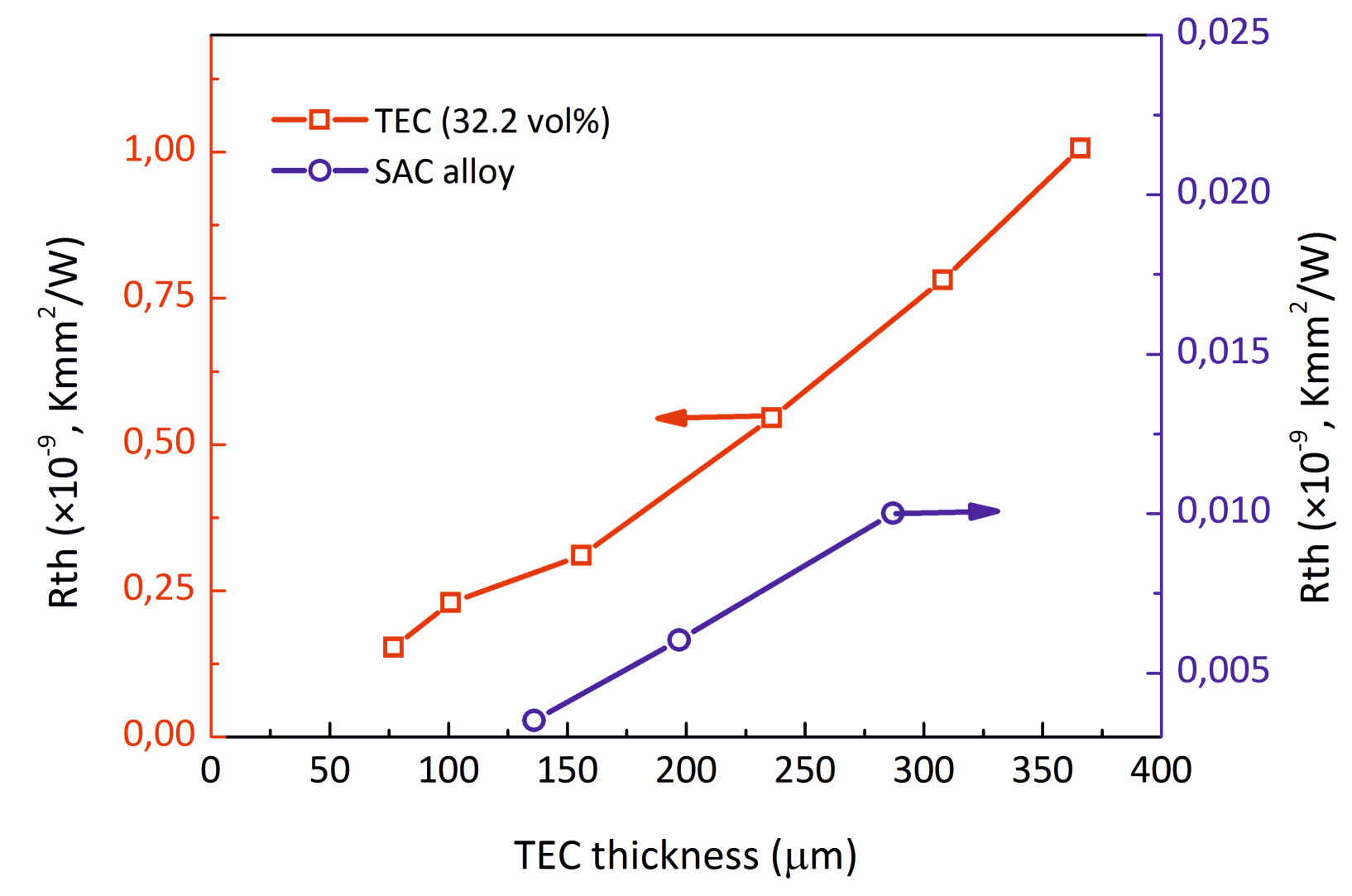


Figure S8. The Xenon flash test samples of TECs coated with graphite layers.


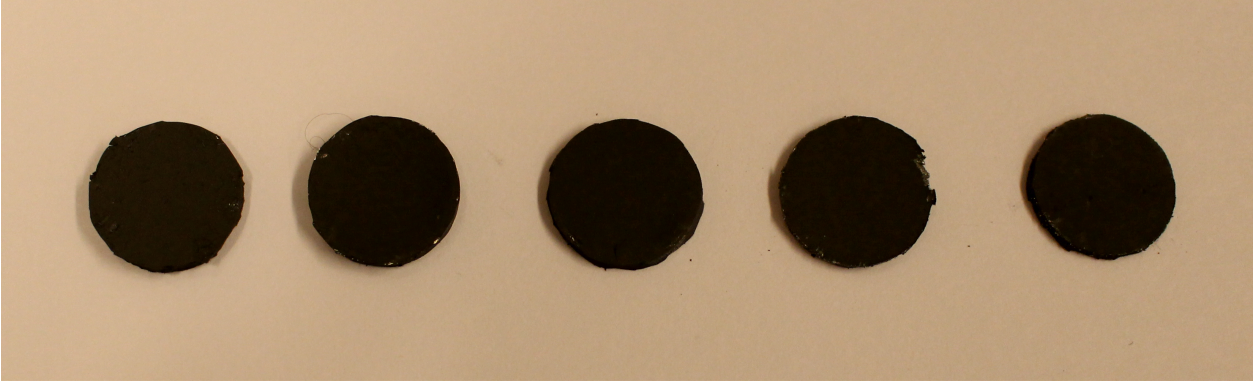


Figure S9. The measurement variation of the thermal conductivity in the Xenon flash test.


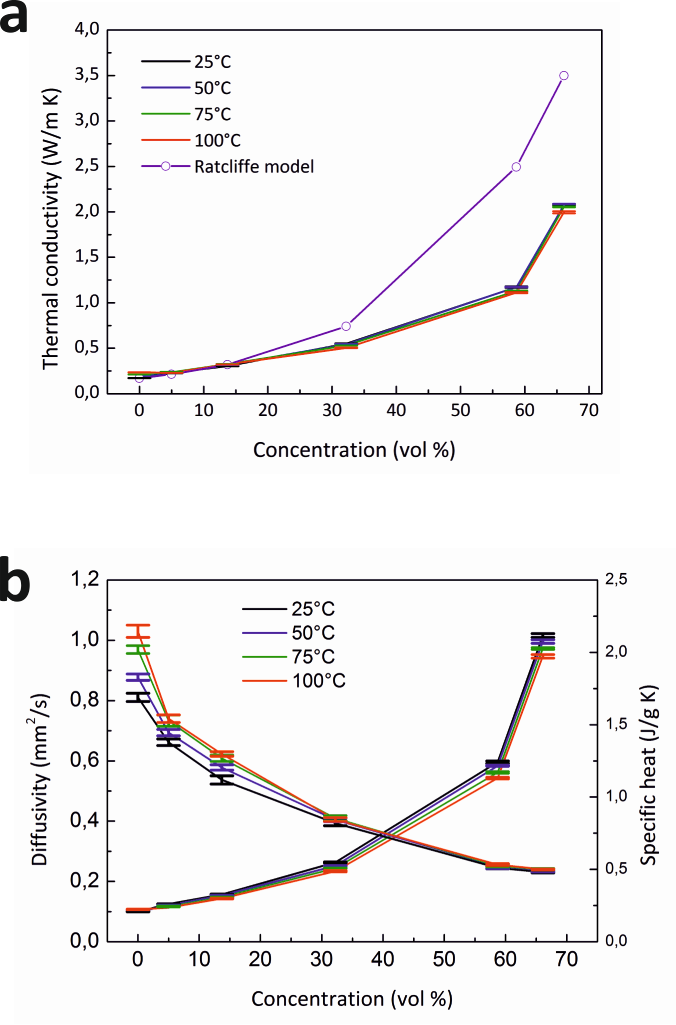

Supplement: Supporting Information [file srep18257-s1.doc]
